# Revision aortic valve replacement with a Perceval sutureless valve 40 years after Björk–Shiley monostrut valve: a case report

**DOI:** 10.1186/s13256-026-06151-3

**Published:** 2026-05-27

**Authors:** Yasuhiro Matsuda, Tadaaki Koyama

**Affiliations:** Department of Cardiovascular Surgery, Kansai Medical University, 2-5-1 Shinmachi, Hirakata, Osaka Japan

**Keywords:** Björk–Shiley, Monostrut, Revision aortic valve replacement, Perceval, Sutureless valve

## Abstract

**Background:**

The Björk–Shiley monostrut (BS-M) valve, a tilting-disc mechanical prosthesis introduced to improve durability and reduce structural failure, has demonstrated excellent long-term outcomes in the aortic position. Although structural valve deterioration is rare, non-valvular complications such as pannus formation can necessitate reoperation. We report a rare case of revision aortic valve replacement 40 years after BS-M implantation due to subaortic stenosis caused by pannus formation, utilizing a Perceval sutureless valve to minimize operative risk in the context of impaired cardiac function.

**Case presentation:**

An 80-year-old Japanese man with a BS-M valve implanted in the aortic position 40 years earlier presented with syncope and progressive heart failure. He had a history of bradycardic atrial fibrillation and had been managed medically for heart failure over the preceding seven years. Transthoracic echocardiography revealed severe aortic stenosis, moderate mitral stenosis, and moderate tricuspid regurgitation, with a significantly reduced ejection fraction. Although the BS-M valve itself exhibited no mechanical damage or disc dysfunction intraoperatively, circumferential pannus formation beneath the prosthesis caused significant subvalvular stenosis. The patient underwent aortic valve re-replacement with a Perceval™ sutureless valve, mitral valve replacement, tricuspid annuloplasty, and left atrial appendage closure. The choice of a sutureless valve was made to reduce myocardial ischemia time. Postoperatively, the patient experienced aspiration pneumonia requiring reintubation; however, he recovered and was transferred to rehabilitation on postoperative day 21. Follow-up echocardiography revealed satisfactory valve hemodynamics and improved ventricular function, with no evidence of prosthetic valve dysfunction. At 8-month follow-up, the patient remained well.

**Conclusions:**

This case highlights the exceptional structural durability of the BS-M valve even after four decades. Nonetheless, non-valvular complications such as pannus formation may necessitate reoperation. Long-term surveillance remains essential for early detection of functional decline, even in patients with structurally intact prosthetic valves.

## Background

The Björk–Shiley (BS) valve, a tilting-disc mechanical prosthesis, was introduced into clinical practice in 1969 and subsequently used worldwide [1]. The original valve utilized a Delrin disc, which was later replaced with pyrolytic carbon due to its durability [2]. Pyrolytic carbon has since become the standard material used in modern mechanical heart valves. Subsequently, the convexo-concave (C-C) model was developed to reduce transvalvular pressure gradients. Despite this modification, reports of strut fractures prompted a transition to the monostrut design, in which the strut and ring are manufactured as a single titanium unit, significantly improving structural integrity [3]. Moreover, with respect to durability, several cases of structural failure have been reported in the mitral position, likely due to the higher transvalvular pressure gradient during valve closure compared to the aortic position [4, 5]. As such, reoperations for BS valves have been reported in the context of Delrin models or mitral position implantation due to disc fracture. In contrast, reoperation due to disc fracture is extremely rare for BS monostrut (BS-M) valve in the aortic position.

We report a case of subaortic stenosis caused by pannus formation 40 years after aortic valve replacement with a BS-M valve, in which the aortic valve was successfully replaced using a sutureless valve (Perceval™, Corcym, Saluggia, Italy).

## Case presentation

An 80-year-old Japanese man had undergone aortic valve replacement with a BS-M valve 40 years earlier. For the past seven years, he had been receiving medical treatment for bradycardic atrial fibrillation and chronic heart failure. Transthoracic echocardiography (TTE) revealed aortic stenosis (AS) with a maximum velocity (Vmax) of 3.87 m/seconds, a peak pressure gradient (pPG) of 60.0 mmHg, a mean pressure gradient (mPG) of 35.0 mmHg, and an aortic valve area (AVA) of 1.12 cm^2^. Three months prior to admission, the patient was transported to our hospital by ambulance due to syncope. Laboratory tests revealed worsening renal function and exacerbation of heart failure (BUN 36 mg/dL, creatinine 1.63 mg/dL, estimated glomerular filtration rate 32 mL/minutes, and NT-proBNP 27,820 pg/mL). TTE demonstrated severe AS and moderate mitral stenosis, with a left ventricular end-diastolic/systolic diameter of 66.1/53.8 mm and an ejection fraction (EF) of 29.1%. Aortic valve findings included a Vmax of 3.52 m/seconds, pPG of 49.5 mmHg, mPG of 32.3 mmHg, and an AVA of 0.87 cm^2^. Mitral valve findings showed a Vmax of 2.47 m/ seconds, pPG of 24.3 mmHg, mPG of 9.8 mmHg, and a mitral valve area of 0.98 cm^2^. Tricuspid regurgitation pressure gradient was 41.9 mmHg, with moderate tricuspid regurgitation. Based on these findings and the presence of New York Heart Association (NYHA) class III heart failure symptoms, surgical intervention was indicated.

**Fig.1:**
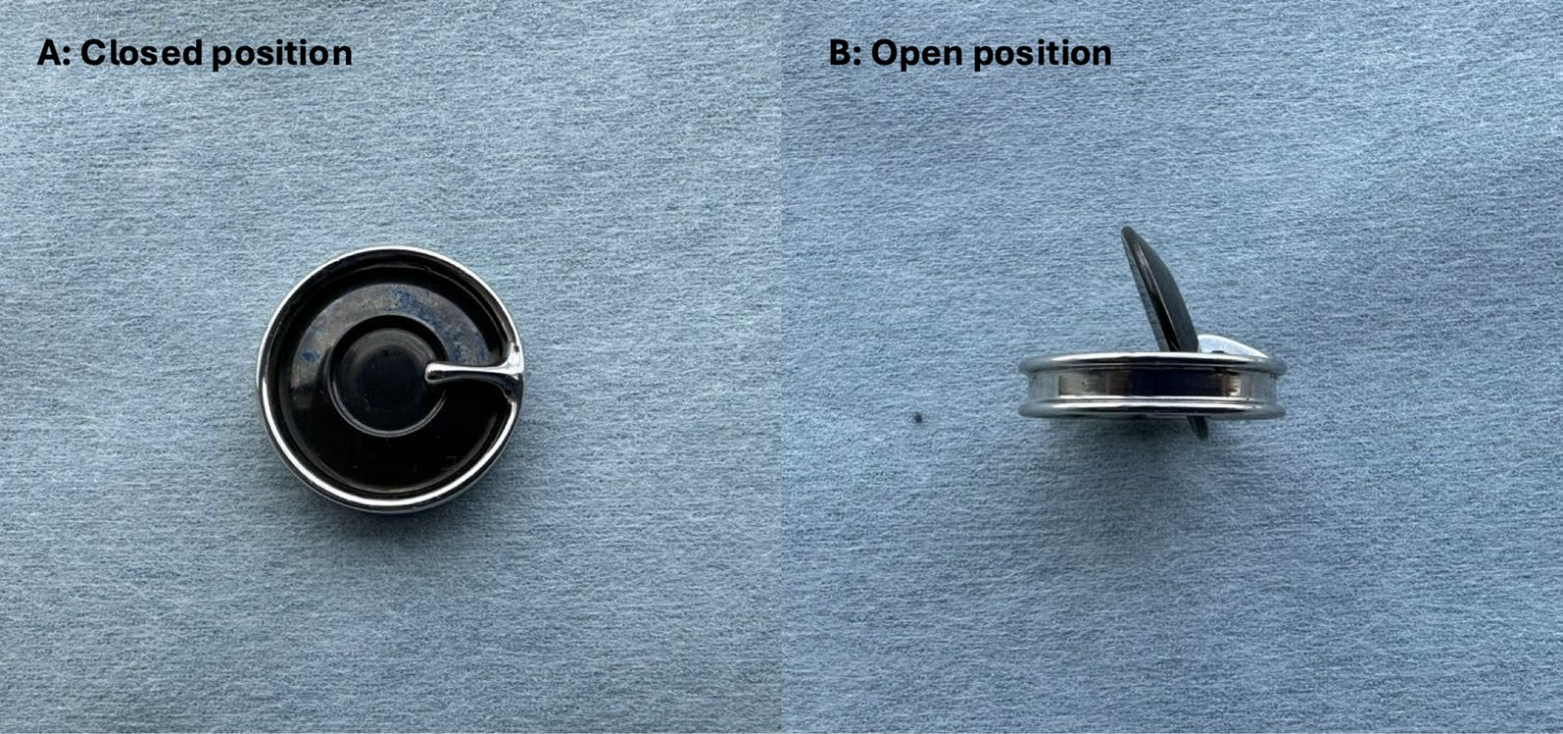
The explanted Björk–Shiley monostrut valve. Leaflet motion was preserved without restriction

The patient underwent aortic valve re-replacement with a Perceval sutureless valve, size L; mitral valve replacement with an Epic™ Mitral Stented Tissue Valve, 31 mm (Abbott, St. Paul, MN, USA); tricuspid annuloplasty with a Tailor™ Flexible Annuloplasty Band, 27 mm (Abbott, Chicago, IL, USA); and left atrial appendage closure. The total operative time was 367 minutes, with a cardiopulmonary bypass time of 250 minutes and an aortic cross-clamp time of 149 minutes. The tilting disc valve showed no mechanical damage, and leaflet motion was intact (Fig. 1). Nevertheless, the tissue was observed circumferentially beneath the aortic prosthetic valve, extending along the entire subvalvular region and resulting in subvalvular stenosis. Pathological examination revealed that the tissue was pannus, and no thrombus was found.

The patient was extubated 22 hours postoperatively and was transferred out of the intensive care unit (ICU) on postoperative day (POD) 4. Catecholamine administration was discontinued on POD 6. On POD 9, he was reintubated due to aspiration pneumonia and required readmission to the ICU. He was successfully extubated on POD 11, and his respiratory condition stabilized on room air after a few days of oxygen therapy. There were no residual respiratory complications or abnormalities. He was transferred to a rehabilitation facility on POD 21. He remains free from recurrent syncope and is being followed as an outpatient with NYHA class II symptoms at 8 months after surgery. Postoperative TTE showed no significant abnormalities, with improved left ventricular size and function (EF of 34%, trivial aortic regurgitation, Vmax of 2.01 m/seconds, pPG of 16 mmHg, mPG of 7.0 mmHg, and an AVA of 1.53 cm^2^).

## Discussion and conclusions

The BS valve, introduced in 1969, is a tilting-disc prosthesis with a single leaflet [1]. The initial model used Delrin, a polyacetal resin, and it was prone to moisture absorption. This property led to disc warping and deformation, raising concerns about long-term durability [6]. Several reports have described cases requiring reoperation after long-term implantation due to disc material degradation [5, 6]. In 1971, the disc material was changed to pyrolytic carbon, which improved the valve’s durability. A spherical disc model with a radiopaque marker was introduced in 1975, as Delrin was radiolucent [7]. In 1976, the convexo-concave (C-C) model was developed to reduce transvalvular pressure gradients. In response to reports of strut fractures observed in the C-C valve, the design was revised in 1982 to develop the monostrut model, integrating the strut and ring to improve structural durability [3].

Regarding the long-term outcomes of the Björk–Shiley (BS) valve, Shinoka et al. reported in 1987 that the rate of re-replacement was higher for Delrin valves implanted in the mitral position [4]. Soofi et al. reported a case in which a Delrin-based Björk–Shiley valve remained functionally intact 42 years after aortic valve replacement [8]. In addition, Hirai et al. compared Delrin and spherical disc models 20 years postoperatively and noted that reoperations due to prosthetic valve dysfunction occurred more frequently in the Delrin group after 15 years [9]. There have also been reports of sudden disc fracture occurring more than 40 years after implantation of a Delrin valve. These cases involved valves placed in the mitral position, which may be attributable to the higher transvalvular pressure gradients during valve closure compared to those in the aortic position [5, 6]. Ahn et al. reported that in the 20-year follow-up of patients with BS-M valves, the incidence of valve-related damage and thromboembolic events was comparable to that observed with other mechanical valves [10]. Moreover, no mechanical failure has been reported with the monostrut model, supporting its long-term structural reliability and safety.

Taken together, the Björk–Shiley valve is recognized for its favorable long-term durability, with valve-related complications being exceedingly rare. Nonetheless, prosthetic valve failure may occur in cases involving the less durable Delrin material or when implanted in the mitral position, where transvalvular pressure gradients are inherently higher. In contrast, pyrolytic carbon valves exhibit excellent structural integrity, and reoperations in such cases have been attributed to non-valvular causes. Mariano et al. described a case requiring reoperation due to prosthesis–patient mismatch [11]. Likewise, Takagi et al. reported a case in which a Bentall procedure was performed for annular dilation 42 years after aortic valve replacement [12]. As in our case, the prosthetic valve itself was entirely intact, and the durability of the pyrolytic carbon model remained excellent even after more than 40 years. Accordingly, reoperations involving BS-M valves in the aortic position are infrequent, with no structural deterioration, thrombus formation, or pannus formation observed in the prosthetic valve.

In the present case, although there was no structural deterioration of the BS-M valve, subvalvular pannus formation led to subaortic stenosis. As the patient also required mitral and tricuspid valve procedures, a sutureless valve was selected for aortic valve replacement to minimize the duration of myocardial ischemia. Given the patient’s impaired cardiac function, the use of the sutureless valve, which allows for reduced cardiopulmonary bypass and aortic cross-clamp durations, was considered an appropriate treatment option.

In conclusion, the BS-M valve may require reintervention due to non-valvular causes, despite its structural durability. Therefore, careful and long-term follow-up is essential.

## Data Availability

The data supporting the conclusions of this case report are included within the article. No additional datasets are available.

## Abbreviations

AS: Aortic stenosis
AVA: Aortic valve area
BUN: Blood urea nitrogen
BS: Björk–Shiley
BS-M: Björk–Shiley monostrut
ICU: Intensive care unit
NT-proBNP: N-terminal pro-B-type natriuretic peptide
POD: Postoperative day
pPG: Peak pressure gradient
mPG: Mean pressure gradient
TTE: Transthoracic echocardiography
Vmax: Maximum velocity
